# Acute Intake of Sucrose but Not of the Intense Sweetener Sucralose Is Associated with Post-Prandial Endotoxemia in Healthy Young Adults—A Randomized Controlled Trial

**DOI:** 10.3390/nu15184038

**Published:** 2023-09-18

**Authors:** Raphaela Staltner, Victor Sánchez, Ina Bergheim, Anja Baumann

**Affiliations:** Department of Nutritional Sciences, Molecular Nutritional Science, University of Vienna, 1090 Vienna, Austria; raphaela.staltner@univie.ac.at (R.S.); victor.sanchez@univie.ac.at (V.S.); ina.bergheim@univie.ac.at (I.B.)

**Keywords:** sucrose, sucralose, intense sweetener, intestinal permeability

## Abstract

Sugar-rich diets, but also the use of intense sweeteners, may alter intestinal barrier function. Here, we assessed the effect of sucrose and sucralose on post-prandial endotoxemia in a randomized placebo-controlled single-blinded crossover-designed study. Following a 2-day standardization of their diet, healthy men and women received a beverage containing either sucrose, sucralose (iso-sweet) or an isocaloric combination of sucralose + maltodextrin. Plasma endotoxin levels were measured after consumption of the respective beverages. Moreover, the effect of sucrose and sucralose on intestinal permeability was assessed in Caco-2 cells and ex vivo in an everted gut sac model. The nutritional standardization recommended by nutrition societies was associated with a significant decrease in plasma endotoxin levels. The intake of the sucrose-sweetened beverage resulted in a significant increase in plasma endotoxin levels while being unchanged after the intake of sucralose-sweetened beverages. In Caco-2 cells, the challenge with sucrose but not with sucralose significantly increased the permeation of the bacterial endotoxin across the cell monolayer. Xylose permeation in small intestinal everted tissue sacs was significantly higher upon the challenge with sucrose while remaining unchanged in sucralose-challenged sacs. Our data suggest that an acute intake of physiologically relevant amounts of sucrose but not of sucralose can result in post-prandial endotoxemia.

## 1. Introduction

Despite ever-increasing weight reduction intervention programs worldwide, the number of overweight and obese individuals is still increasing, as are instances of metabolic diseases and cancer [1]. In this context, changes in intestinal barrier function and the composition of intestinal microbiota may play an important role [2]. Indeed, recent findings suggest that changes in intestinal barrier function may lead to an elevated translocation of bacterial toxins such as lipopolysaccharide into the blood stream of the host (=endotoxemia). This development activates the Toll-like receptor 4 signaling cascade, resulting in an increase in pro-inflammatory markers leading to a low-grade inflammation [3]. In 2007, Cani et al. defined metabolic endotoxemia as a state induced by dietary patterns [4]. Several dietary factors have been suggested to be critical in the development of metabolic endotoxemia, including saturated fats and sugars like fructose (for overview [3,5]). Indeed, while the World Health Organization (WHO) recommended in 2015 that the intake of free/added sugars should constitute ≤ 10% of total daily energy intake (E%) [6], sugar intake is still markedly high in many industrialized countries. For instance, in Austria and Germany, added sugar contributes to ~14% of energy intake (E%) [7], while in the Netherlands it is 12 E% [8].

Sugar-sweetened beverages have been identified as one major source of added sugars across all age groups in several epidemiological studies and countries [9,10]. Furthermore, epidemiological studies assessing the effects of substituting sugar-sweetened beverages with beverages sweetened with low- or no-calorie sweeteners suggest that this exchange was associated with small improvements in body weight and cardiometabolic risk factors and was almost as effective as a substitution with water [11,12]. In contrast, results of studies in model organisms suggest that the high intake of low- or no-calorie sweeteners, especially over an extended period of time, might promote adverse effects, including excessive weight gain, insulin resistance and alterations to intestinal microbiota composition [13,14]. However, the results of these studies also suggest that the effects of different sweeteners may vary considerably and may even depend on whether they are consumed solely or in combinations/mixtures, as it is often found in commercially available intense sweeteners (for overview see [15,16]).

Sucralose, being frequently used either solely or in combination in beverages [17], is a no-calorie sweetener with a sweetness about 600 times higher than sucrose (with an acceptable daily intake of 15 mg/kg body weight (EFSA) [18] and 5 mg/kg body weight (FDA) [19]). In human intervention studies, it was shown that sucralose, when consumed regularly in elevated concentrations for weeks, may impair glucose tolerance [20]. Recently, it was shown in mice that an intake of sucralose may reduce T cell proliferation and differentiation [21]. However, whether an acute intake of sucralose also affects intestinal barrier function and subsequently the translocation of bacterial endotoxin, which may also affect immune response, has not yet been clarified.

Starting from this background, the aim of the present single-blinded crossover-designed study was to assess the effects of an acute intake of sucralose in physiological doses compared to an iso-sweet intake of sucrose on post-prandial endotoxemia in healthy young adults.

## 2. Materials and Methods

### 2.1. Study Participants

This randomized controlled prospective human intervention study in crossover design was approved by the Ethics Committee of the University of Vienna (reference number: 00585) and was carried out in accordance with the ethical standards laid down in the Declaration of Helsinki. Originally, it was planned to investigate the effect of an acute (only once) and a two-day long intake of sucralose and sucrose on post-prandial endotoxemia in healthy subjects. Due to the COVID-19 pandemic situation and the associated restrictions, we were not able to conduct the study as originally planned and therefore focused on the acute intervention only. The study is registered at ClinicalTrials.gov (NCT04788680) and obtained approval to be carried out between 2021 and 2022. Sample size was calculated based on previous studies [22]. A total of 18 healthy, normal-weight (BMI > 18.5 kg/m^2^ or <24.9 kg/m^2^), non-smoking people were enrolled in this study after giving written informed consent. In total, 11 participants finished all interventions whereas 7 participants did not start and dropped out of the study due to corona infection (*n* = 2), personal reasons (*n* = 3) or moving to another city (*n* = 2). At the time of enrollment, none of the participants (1) followed a special diet or had (2) food malabsorption or (3) a history of chronic diseases of the gastrointestinal tract. Additionally, none of the subjects reported drinking more than a moderate amount of alcohol (>10 g/day for women; >20 g/day for men). 

### 2.2. Intervention Study

**Figure 1:**
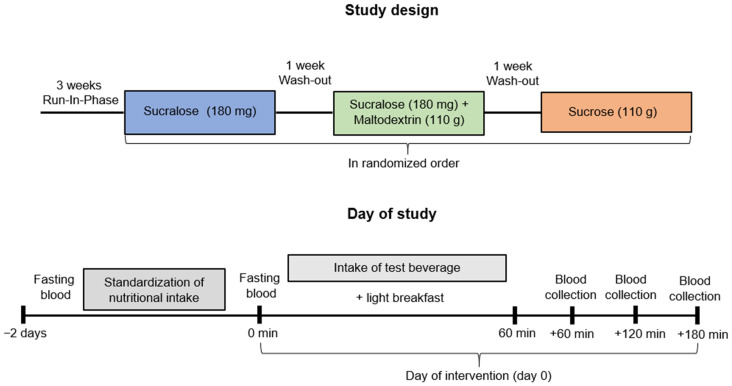
Design of the randomized controlled single-blinded crossover-designed human intervention study.

The study design is summarized in Figure 1 and aimed to assess the effect of different sweetened beverages on post-prandial endotoxemia (primary outcome) and blood lipid profile (secondary outcome) in healthy young adults. Caloric intake of all participants was assessed in two 24-h recalls (one weekday and one weekend day). Three weeks before the study, participants were asked to refrain from the consumption of intense sweeteners. Participants were randomly assigned (block randomization via online calculator) to the study interventions. Thereafter, all participants were standardized in their nutrition based on their caloric intake according to the recommendations of the German, Austrian and Swiss Nutrition Societies (D-A-CH) for two days prior to each intervention day. Nutritional intake and standardization of nutrition were calculated via the computer software EBISpro (Version 2011, Willstätt, Germany). Food, beverages and recipes were provided by the study team at the University of Vienna. The study was conducted in crossover design and all participants consumed the three different beverages in a randomized order. On the day of intervention (day 0), fasting blood was drawn before the participants were asked to consume the different study beverages (1 L) and a light breakfast (a roll and 10 g butter) within 60 min. The beverages contained either sucrose (110 g), the intense sweetener sucralose (180 mg) in an iso-sweet amount or the isocaloric combination of the intense sweetener sucralose (180 mg) + maltodextrin (110 g) in carbonated fountain water (1 L total volume) (see Table 1). Additional blood samples were collected after 60, 120 and 180 min. The three interventions were separated by a wash-out period of at least one week. Fasting blood was drawn before and after each nutritional standardization.

### 2.3. Anthropometry, Blood Pressure and Metabolic Parameters

At the beginning and over the course of the study, anthropometric data and blood pressure were determined. Blood lipids were measured using a commercially available measuring instrument (Swiss Point of Care, LJ IJsselstein, The Netherlands). Blood sugar levels were assessed in capillary blood obtained from the fingertip by using a finger-stick glucometer before and after standardization and during the intervention.

### 2.4. Bacterial Endotoxin

To assess the effect of the standardized nutrition as well as of the different beverages on bacterial endotoxin levels in plasma of participants over time, a limulus amebocyte lysate assay was used (Charles River, Ecully, France) as detailed previously [23]. In addition, to determine endotoxin levels in cell culture experiments (see below), a SEAP reporter HEK293 cell assay (Invivogen, Toulouse, France) activated by Toll-like receptor 4 ligands was used as detailed previously [24]. 

### 2.5. Caco-2 Cells In Vitro Experiments

Differentiated Caco-2 cells (ACC 169, DSMZ, Braunschweig, Germany) were grown in trans-wells by using DMEM medium containing 10% fetal bovine serum (Pan-Biotech GmbH, Aidenbach, Germany) and 100 µg/mL streptomycin and 100 U/mL penicillin (Pan-Biotech GmbH, Aidenbach, Germany) in a 5% carbon dioxide atmosphere. After reaching confluency, cells were treated with sucralose (0.016 mM) or sucrose (10 mM) in the apical compartment of the trans-well for 2 h. Lipopolysaccharide (LPS, 100 ng/mL) was added to the apical side after 2 h for another 1 h and bacterial endotoxin levels were measured in the media of the basolateral compartment.

### 2.6. Enzyme-Linked Immunosorbent Assay (ELISA)

Intestinal fatty acid binding protein (iFABP) concentrations were analyzed in cell culture supernatant of the apical side of the Caco-2 cell trans-well model using a commercially available ELISA kit (Bio-Techne Corp., Minneapolis, MN, USA).

### 2.7. Ex Vivo Everted Gut Sac Experiments and Xylose Permeation Measurement

Small intestine (*n* = 6) from naïve female C57BL/6J mice (Janvier Labs, Le Genest-Saint-Isle, France) were collected, rinsed with PBS and everted with a rod as previously described [25]. Small intestinal tissue was cut into equal pieces and the ends of the gut sacs were ligated with a knot. These everted gut tissue sacs were filled with 1× Krebs-Henseleit buffer supplemented with 0.2% bovine serum albumin and incubated in 1× Krebs-Henseleit buffer (Ctr) supplemented with either sucrose (10 mM) or sucralose (0.016 mM) for 55 min at 37 °C in a gaseous 95% O_2_/5% CO_2_ atmosphere. To determine intestinal permeability, everted gut tissue sacs were incubated in a solution containing 0.1% xylose +/− sucrose or sucralose for additional 5 min. Xylose concentration in liquids of the everted gut sacs was measured using an assay based on phloroglucinol as previously described [26]. 

### 2.8. Statistical Analysis

Data are presented as means ± SEM. To test for normality, a Shapiro–Wilk normality test was performed. Grubb’s test was used before statistical analysis to identify outliers. To assess the effects between two paired groups, a paired *T*-Test was used. Ordinary one-way ANOVA with Tukey’s multiple comparison test was used to determine statistically significant differences between interventions. Two-way ANOVA with Tukey’s multiple comparison test was used to determine statistically significant differences within an intervention over time. All data were analyzed with GraphPad Prism software version 7 (GraphPad Prism Software Inc., San Diego, CA, USA). Significance was considered at a *p*-value < 0.05.

## 3. Results

### 3.1. Baseline Characteristics and Nutritional Standardization

Of the 18 normal-weight, healthy men and women enrolled in the study, 11 participants finished the study and were analyzed. Seven participants did not start the study as they dropped out of the study due to corona infections (*n* = 2), moving to another city (*n* = 2) or personal reasons (*n* = 3). The baseline characteristics of the analyzed participants, including their anthropometry, routine laboratory parameters and nutritional intake, are summarized in Table 2 and Table 3. After assessing their health status and nutritional intake, participants were asked to consume an isocaloric, nutritionally balanced diet following the recommendation of the D-A-CH [27]. During the two days of standardization, when all foods and beverages besides plain water were provided by the study team, anthropometric parameters remained unchanged. For most participants, the standardization of nutritional intake was associated with a lower intake of total fat (−~14% (E%)) and protein (−~0.6 g/kg body weight) and an increase in total fruit and vegetable intake, but also carbohydrate intake (+~17% (E%)) and fiber (+~8 g/day) (see Table 3), serum triglyceride levels (−~11 mg/dL) and total cholesterol (−~11 mg/dL) as well as HDL (−~2 mg/dL) and LDL (−~7 mg/dL) cholesterol either decreased by trend (for triglycerides) or significantly (for total cholesterol and HDL and LDL cholesterol, see Table 4). Furthermore, systolic blood pressure and bacterial endotoxin levels in the serum were also significantly lower after the two days of nutritional standardization (see Table 4).

### 3.2. Effect of an Acute Intake of Sucralose, Sucrose and an Isocaloric Combination of Sucralose and Maltodextrin, Respectively, on Post-Prandial Bacterial Endotoxin Levels in Blood

**Figure 2:**
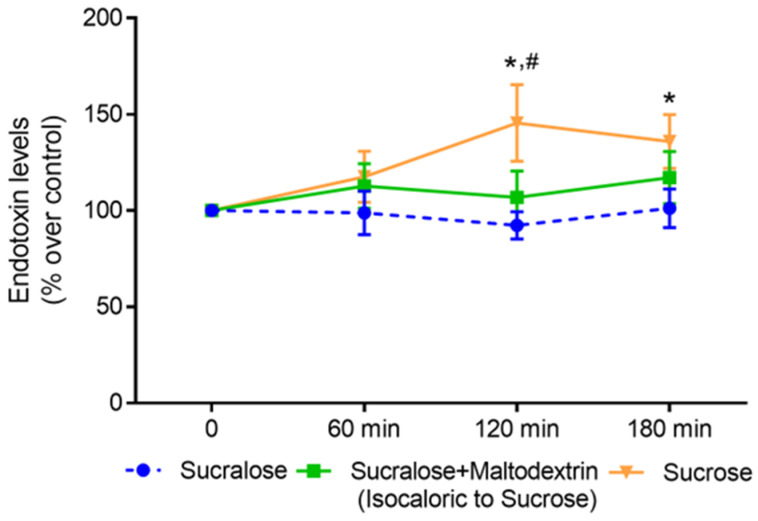
Effect of a beverage containing either sucrose, sucralose or sucralose + maltodextrin on bacterial endotoxin levels in peripheral blood 60, 120 and 180 min after the consumption of the respective beverage. Values are means ± SEM, *n* = 11 except for intervention sucralose + maltodextrin: *n* = 10. * *p* < 0.05 increase in endotoxin levels 120 and 180 min after consumption of the beverage containing sucrose compared with baseline, # *p* < 0.05 increase in endotoxin levels 120 min after consumption of the beverage containing sucrose compared to the beverage containing sucralose.

After the intake of the sucrose-sweetened beverage, plasma endotoxin levels increased significantly when compared with baseline levels (120 min: +~45%, 180 min: +~36% compared with baseline, Figure 2). A similar effect was not found when subjects consumed the sucralose-sweetened beverage or the beverage sweetened with sucralose + maltodextrin (isocaloric control). Furthermore, 120 min after the intake of the sucrose-sweetened beverage, bacterial endotoxin levels in peripheral plasma were significantly higher than they were after the consumption of the sucralose-sweetened beverage (Figure 2). 

### 3.3. Effect of Sucralose and Sucrose on Bacterial Endotoxin Permeation and Protein Levels of iFABP in Differentiated Caco-2 Cells

**Figure 3:**
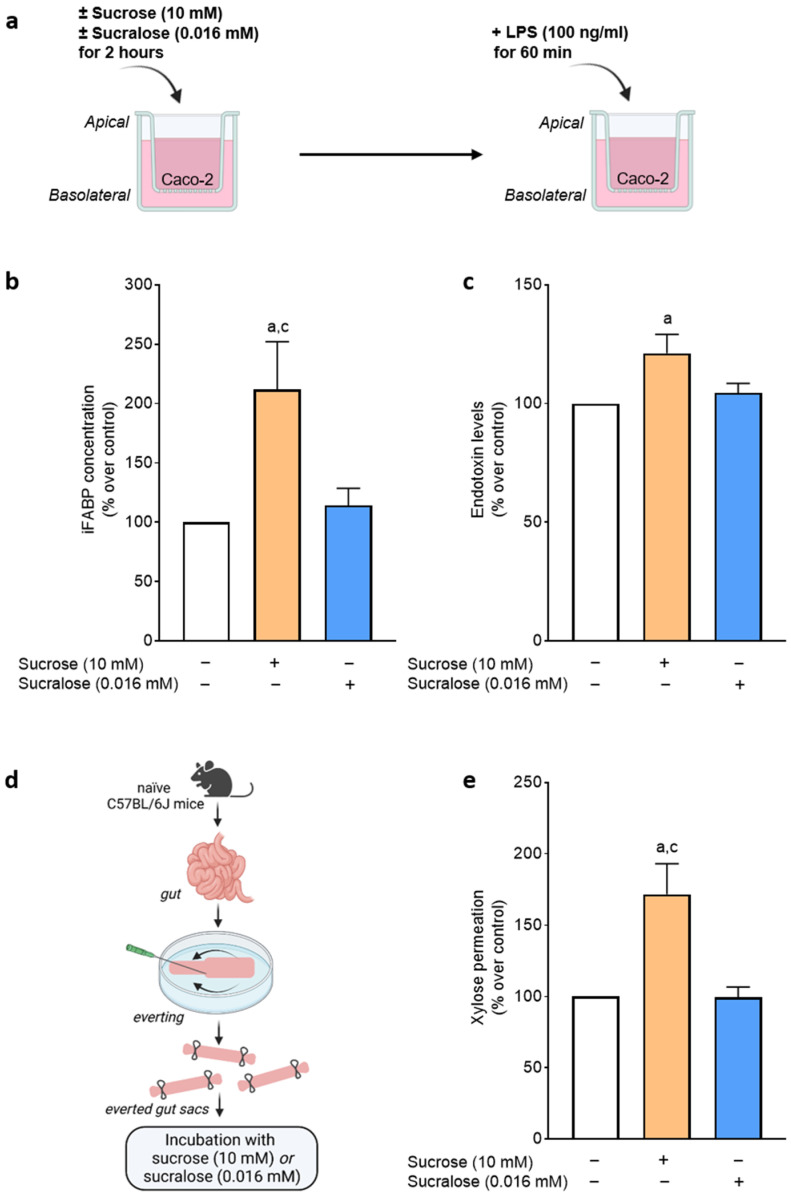
(**a**) Graphical illustration of the experimental cell culture setup. (**b**) Effect of sucralose (0.016 mM) and sucrose (10 mM) on intestinal fatty acid binding protein (iFABP) concentration in the apical compartment of the trans-well system after 2 h. (**c**) Endotoxin levels in the basolateral compartment after additional 60 min of lipopolysaccharide (LPS) treatment (100 ng/mL). (**d**) Graphical illustration of the everted gut sac model. (**e**) Xylose permeation of everted gut sacs incubated with sucralose (0.016 mM) or sucrose (10 mM). Values are means ± SEM, for (**b**,**c**): *n* = 4–5; for (**e**): *n* = 6. ^a^ *p* < 0.05 compared with control, ^c^ *p* < 0.05 compared with sucralose. Graphical illustration (**d**) was created with BioRender.com.

To further assess if the increase in bacterial endotoxin levels found after the intake of sucrose was related to changes in intestinal barrier function and to compare these effects with those of sucralose, a model of the intestinal barrier, e.g., differentiated Caco-2 cells grown on trans-well inserts was employed in which cells were incubated with sucrose or sucralose in iso-sweet, physiological doses (see Figure 3 for experimental setup). The addition of sucralose to the apical side of the trans-wells prior to the apical exposure to bacterial endotoxin had no effect on bacterial endotoxin levels in the basolateral compartment of the well system. In contrast, bacterial endotoxin levels were significantly higher in the basolateral compartment of the trans-wells when sucrose was added to the apical compartment of the trans-well system prior to the exposure to bacterial endotoxin. In line with these findings, the iFABP concentration, shown before to be indicative of intestinal barrier disruption [28], also only increased in apical media of cells challenged with sucrose while remaining at the level of controls in cells exposed to sucralose for 2 h (Figure 3).

### 3.4. Effect of Sucralose and Sucrose on Markers of Intestinal Barrier Function in Small Intestinal Everted Tissue Sacs of C57BL/6J Mice

When challenging small intestinal everted tissue sacs with sucrose, the permeation of xylose significantly increased compared with tissue sacs only incubated with 1× Krebs–Henseleit buffer. In contrast, the intestinal permeation of xylose was not altered when tissue sacs were challenged with sucralose (Figure 3).

## 4. Discussion

Intense sweeteners widely used in human nutrition as substitutes for sugars like sucrose or high-fructose corn syrup may have beneficial effects with regard to body weight gain and the development of some non-communicable diseases [29]. However, knowledge of their effects on the intestine is still limited. In the present study, subjects consumed, once, either 1 L of a beverage sweetened with sucrose or sucralose or a beverage isocaloric to the sucrose beverage but sweetened with sucralose to assess the effects of these different beverages on post-prandial endotoxemia. While the serving size of sugar-sweetened and intense sweetener-sweetened beverages varies considerably between countries [30], the consumption of 1 L of a sucrose-sweetened beverage may be considered to be rather high. Still, epidemiological studies suggest that in many areas around the world, the average daily intake of sugar derived from sugar-sweetened beverages ranges between 100 and 300 g [9] and it has been shown that in subgroups, especially in adolescents and young adults, the intake of sugar-sweetened beverages may even exceed 1 L [31,32]. Here, the intake of the sucrose-sweetened beverage resulted in a significant increase in bacterial endotoxin levels (~45% after 120 min) in the peripheral blood while no alike changes were found after the intake of either the sucralose-sweetened beverage or the sucralose-sweetened beverage enriched with maltodextrin. In line with these findings, neither the exposure of differentiated Caco-2 cells grown on trans-wells nor that of small intestinal tissue sacs to sucralose was associated with any changes in the translocation of bacterial endotoxin from the apical to the basolateral compartment or changes in iFABP protein levels in the cell culture medium, whereas these markers were significantly altered when cells were exposed to sucrose. It has been shown before by us and others that the intake of certain foods like emulsified milk fat [33,34], fructose [35] and high-fructose corn syrup [22,36] may result in an increase in bacterial endotoxin levels. For instance, after the intake of ~160 g of fructose for 4 days, bacterial endotoxin levels increased ~1.3-fold in the peripheral blood of healthy subjects. In contrast, similar effects were not found when consuming ~160 g of glucose [22]. These findings together with studies in mice [37] suggest that the increase in endotoxin levels related to the intake of sucrose may depend upon its fructose content. Recent studies in humans and rodents suggest that fructose may not only alter intestinal microbiota composition but may also alter intestinal nitric oxide (NO) homeostasis in small intestinal tissue. These alterations may subsequently lead to a loss of tight junctions and the increased permeation of bacterial endotoxin through so-far-not-fully-understood mechanisms (for overview [5]). Somewhat contrasting with the findings of our study, rodents fed a high-fat diet enriched with elevated amounts of sucrose (10% drinking solution) and rodents fed a high-fat diet with sucralose (1.5% drinking solution) for 4 months showed an enrichment of bacterial genes involved in the synthesis of LPS and an increase in bacterial endotoxin levels in peripheral blood [38]. The differences between our study and that of Sanchez-Tapia et al. [38] regarding the effects of sucralose might have resulted from the differences in species (humans vs. rodents) and the duration of the treatment (in the present study, once vs. several weeks). Taken together, our results suggest that an acute intake of sucrose in concentrations found in “normal,” sweet-tasting beverages but not the intake of sucralose may result in post-prandial endotoxemia in healthy young adults.

In line with our previous findings [22], the nutritional standardization of subjects for only 2 days was associated with a significant decrease in bacterial endotoxin levels in their plasma as well as a decrease in diastolic blood pressure, total cholesterol and LDL cholesterol and triglyceride levels in their blood. The nutritional intake of the healthy, normal-weight young adults enrolled in the present study was rather high in fat and protein while being low in carbohydrates and fiber. A diet high in fat and low in fiber has been suggested to be especially critical in the development of cardiovascular disease [39] whereas a diet rich in fruit and vegetables may bear beneficial effects regarding the risk of hypertension [40,41]. However, it remains to be determined if changes in nutrient composition in general or only in specific compounds found in different foods add to the changes in clinical parameters, e.g., blood pressure, but even more so in bacterial endotoxin levels. 

### Limitations

Our study is not without limitations that need to be considered when interpreting the data. A major limitation of our study is the sample size of only 11 subjects and the focus on young, healthy adults. The results might differ in a larger cohort and in another age group as well as in subjects with metabolic abnormalities, e.g., type 2 diabetes or metabolic dysfunction-associated steatotic liver disease (MASLD). This needs to be assessed in future studies. Another limitation is that the subjects consumed the different beverages only once. Therefore, from the present study, no estimation regarding the long-term effects of an intake of sucralose, especially of large amounts, on intestinal barrier function can be made. Additionally, in the present study, no direct measurements of intestinal permeability, e.g., through a xylose or lactose-mannitol test were included. However, these tests require not only the ingestion of an additional beverage but also urine collection over several hours. As the dietary interventions were rather rigid and participants had to consume a large amount of liquid in a rather short period of time on the day of the study, these additional tests would have resulted in lower compliance and therefore decreased the number of participants even further. 

## 5. Conclusions

In summary, our data suggest that the intake of a sucralose-sweetened beverage in a physiological amount, e.g., 1 L, has no effect on intestinal barrier function in healthy young adults. To our knowledge, this is the first study which examines the effect of an acute intake of sucralose on endotoxin levels in healthy, normal-weight human subjects. However, whether an intake of sucralose over an extended period of time and at higher doses or in individuals suffering from health impairments has similar limited effects on intestinal barrier function needs to be determined in future studies. The results of our study also add further weight to the hypothesis that dietary sugars like sucrose may be critical in the development of intestinal barrier dysfunction suggested to contribute to the development of various metabolic diseases like MASLD and type 2 diabetes [42]. Further studies are needed to determine the molecular mechanisms underlying the effects of sucrose on intestinal barrier dysfunction.

## Figures and Tables

**Table 1:** Composition of breakfast on day of study.

	Sucralose	Sucralose + Maltodextrin	Sucrose
Water (L) containing	1	1	1
Sucrose (g)	-	-	110
Sucralose (mg)	180	180	-
Maltodextrin (g)	-	110	-
Butter (g)	10	10	10
Roll (g)	65	65	65
Energy (kcal)	235.4	681.5	681.5
Carbohydrates (E%)	57.0	85.0	85.0
Fat (E%)	35.0	12.0	12.0
Protein (E%)	8.0	3.0	3.0

E%: percentage of energy.

**Table 2:** Characteristics of healthy male and female participants.

Parameter	Baseline
Gender (m/f)	5/6
Age (years)	26.2 ± 0.8
BMI (kg/m^2^)	21.9 ± 0.5
WHR	0.85 ± 0.01
Blood glucose (mg/dL)	81.4 ± 2.6
Total cholesterol (mg/dL)	172.1 ± 6.8
Triglycerides (mg/dL)	71.9 ± 7.1
HDL (mg/dL)	60.2 ± 2.5
LDL (mg/dL)	98.8 ± 6.9
Blood pressure	
Systolic blood pressure (mm Hg)	118.9 ± 1.8
Diastolic blood pressure (mm Hg)	75.2 ± 2.7
Uric acid (mg/dL)	4.3 ± 0.3
Bilirubin (mg/dL)	2.2 ± 1.7
ALT (U/L)	24.0 ± 1.9
AST (U/L)	24.4 ± 3.4
Alkaline phosphatase (U/L)	59.5 ± 6.3
Gamma-GT (U/L)	14.2 ± 1.6
C-reactive protein (mg/dL)	0.1 ± 0.0

Values are means ± SEM, *n* = 11. BMI: Body Mass Index; WHR: waist-to-hip ratio; HDL: high-density lipoprotein; LDL: low-density lipoprotein; ALT: alanine aminotransferase; AST: aspartate aminotransferase; Gamma-GT: Gamma-glutamyltransferase.

**Table 3:** Nutritional intake of participants before and during the nutritional standardization according to the recommendations of the German, Austrian and Swiss Nutrition societies (D-A-CH) prior to each intervention.

Parameters and Recommendations ^a^	24 h Recall	Standardization
Energy (kcal/day)	2194 ± 112.4	2237 ± 95.4
Carbohydrates (E%) (>50 E%) ^a^	41.7 ± 3.2	59.0 ± 0.3 *
Total polysaccharides (g)	141.8 ± 21.9	202.0 ± 11.5 *
Total vegetable consumption (g) (≥400 g/day) ^a^	253.5 ± 41.7	551.1 ± 18.0 *
Total fruit consumption (g) (≥250 g/day) ^a^	229.8 ± 60.2	373.5 ± 16.0 *
Total fructose ^b^ (g)	30.8 ± 3.9	53.8 ± 1.8 *
Added fructose (g)	14.5 ± 2.5	15.7 ± 1.5
Total glucose ^c^ (g)	30.4 ± 5.1	50.4 ± 1.7 *
Added glucose (g)	17.0 ± 4.2	15.4 ± 1.8
Total sucrose (g)	26.4 ± 3.8	49.7 ± 2.3 *
Added sucrose (g)	15.4 ± 3.4	14.9 ± 1.2
Total added sugar (E%) (<10 E%) ^a^	8.3 ± 1.8	8.3 ± 0.8
Protein (g/kg body weight/day) (0.8 g/kg body weight/day) ^a^	1.4 ± 0.1	0.8 ± 0.0 *
Fat (E%) (30 E% ^d^) ^a^	45.5 ± 4.5	31.3 ± 0.2 *
SFA (E%) (<10 E%) ^a^	14.6 ± 1.4	8.6 ± 0.3 *
Fiber (g) (≥30 g/day) ^a^	27.2 ± 3.0	34.8 ± 1.0 *

Values are means ± SEM, *n* = 11, * *p* < 0.05. E%: percentage of energy; SFA: saturated fatty acids; ^a^ Recommendations where applicable; ^b^ Fructose derived from fruits, vegetables, and sucrose; ^c^ Glucose derived from fruits, vegetables, and sucrose; ^d^ Higher percentages possible with Physical Activity Level > 1.7.

**Table 4:** Effect of the nutritional standardization following the recommendations of the German, Austrian and Swiss Nutrition societies (D-A-CH) on anthropometric and clinical parameters as well as bacterial endotoxin levels in healthy participants.

Parameters	Before Standardization	After Standardization
Body weight (kg)	67.5 ± 2.5	67.6 ± 2.5
BMI (kg/m^2^)	21.9 ± 0.5	22.0 ± 0.5
Blood glucose (mg/dL)	96.5 ± 0.9	97.6 ± 1.4
Triglycerides (mg/dL)	84.2 ± 7.2	72.8 ± 4.6 ^a^
Total Cholesterol (mg/dL)	161.3 ± 12.4	150.6 ± 10.7 *
HDL (mg/dL)	47.9 ± 4.3	45.6 ± 4.1 *
LDL (mg/dL)	96.3 ± 10.8	89.6 ± 9.8 *
Systolic blood pressure (mm Hg)	122.5 ± 3.3	120.5 ± 3.0
Diastolic blood pressure (mm Hg)	79.4 ± 1.8	76.7 ± 1.5 *
Endotoxin (EU/mL)	1.8 ± 0.2	1.5 ± 0.1 *

Values are means ± SEM, *n* = 11, * *p* < 0.05; ^a^ *p* = 0.053. BMI: Body Mass Index; HDL: high-density lipoprotein; LDL: low-density lipoprotein.

## Data Availability

Data are made available upon reasonable request.
